# Soluble Neuropilin-1 Is Elevated in Sepsis and Correlates with Organ Dysfunction and Long-Term Mortality in Critical Illness

**DOI:** 10.3390/ijms25105438

**Published:** 2024-05-16

**Authors:** Philipp Hohlstein, Eileen Schumacher, Samira Abu Jhaisha, Jule K. Adams, Maike R. Pollmanns, Carolin V. Schneider, Karim Hamesch, Katarina Horvathova, Theresa H. Wirtz, Frank Tacke, Christian Trautwein, Ralf Weiskirchen, Alexander Koch

**Affiliations:** 1 Department for Gastroenterology, Metabolic Disorders and Intensive Care Medicine, RWTH-University Hospital Aachen, Pauwelsstraße 30, 52074 Aachen, Germany; phohlstein@ukaachen.de (P.H.); eischumacher@ukaachen.de (E.S.); sabujhaisha@ukaachen.de (S.A.J.); jadams@ukaachen.de (J.K.A.); mpollmanns@ukaachen.de (M.R.P.); cschneider@ukaachen.de (C.V.S.); khamesch@ukaachen.de (K.H.); thwirtz@ukaachen.de (T.H.W.); trautwein.christian@gmx.net (C.T.); 2 Biomedica Slovakia s.r.o., 841 01 Bratislava, Slovakia; katarina.horvathova@bmgrp.sk; 3 Department of Hepatology and Gastroenterology, Charité-Universitätsmedizin Berlin, Campus Virchow-Klinikum (CVK) and Campus Charité Mitte (CCM), Augustenburger Platz 1, 13353 Berlin, Germany; frank.tacke@charite.de; 4 Institute of Molecular Pathobiochemistry, Experimental Gene Therapy and Clinical Chemistry (IFMPEGKC), RWTH-University Hospital Aachen, Pauwelsstraße 30, 52074 Aachen, Germany; rweiskirchen@ukaachen.de

**Keywords:** Neuropilin-1, intensive care unit, critical illness, sepsis, human, inflammation, immune system, prognosis, survival, mortality

## Abstract

Critical illness and sepsis may cause organ failure and are recognized as mortality drivers in hospitalized patients. Neuropilin-1 (NRP-1) is a multifaceted transmembrane protein involved in the primary immune response and is expressed in immune cells such as T and dendritic cells. The soluble form of NRP-1 (sNRP-1) acts as an antagonist to NRP-1 by scavenging its ligands. The aim of this study was to determine the value of sNRP-1 as a biomarker in critical illness and sepsis. We enrolled 180 critically ill patients admitted to a medical intensive care unit and measured serum sNRP-1 concentrations at admission, comparing them to 48 healthy individuals. Critically ill and septic patients showed higher levels of sNRP-1 compared to healthy controls (median of 2.47 vs. 1.70 nmol/L, *p* < 0.001). Moreover, sNRP-1 was also elevated in patients with sepsis compared to other critical illness (2.60 vs. 2.13 nmol/L, *p* = 0.01), irrespective of disease severity or organ failure. In critically ill patients, sNRP-1 is positively correlated with markers of kidney and hepatic dysfunction. Most notably, critically ill patients not surviving in the long term (one year after admission) showed higher concentrations of sNRP-1 at the time of ICU admission (*p* = 0.036), with this association being dependent on the presence of organ failure. Critically ill and septic patients exhibit higher serum concentrations of circulating sNRP-1, which correlates to organ failure, particularly hepatic and kidney dysfunction.

## 1. Introduction

Sepsis is a significant contributor to mortality among hospitalized patients and has been redefined in the Sepsis-3 criteria as a life-threatening host response resulting in organ failure due to infection [1,2]. The global incidence of sepsis was estimated at 48.9 million cases in 2017 (equivalent to 677.5 cases per 100,000 age-standardized population), accounting for 29.5% of admissions to the intensive care unit (ICU) [3,4]. The improvement of outcomes is mainly achieved by early identification and appropriate management in the initial hours after the onset of sepsis. To date, even established biomarkers such as procalcitonin have been unable to accurately identify patients at the onset of sepsis and indicate their prognosis or the expected extent of organ failure [5,6]. Moreover, the mechanisms causing the uncontrolled infection, dysregulated immune response, and development of organ failure are not yet fully understood, necessitating further investigation [7,8,9]. Early exacerbated systemic inflammation in sepsis leads to an alteration of the function and composition of cells involved in acquired immunity, i.e., an apoptotic process resulting in tremendous lymphopenia [10]. The remaining T cells not undergoing apoptosis after sepsis display an anergic or exhausted profile, resulting in decreased T cell proliferation among other functional impairments, likely caused by the massive initial activation in the septic immune response [10]. In fact, lymphocytes and, in particular, T cells, have been described as useful biomarkers for the prediction of prognosis in sepsis and critical illness [6,11,12]. Persistent lymphopenia is also associated with nosocomial infections and an increased susceptibility of patients with sepsis to secondary infections [13,14,15]. A wide range of other biomarkers, including but not limited to, cytokines, non-coding RNAs and microRNAs, membrane receptors, cell proteins, and metabolites, have been studied to stratify the prognosis of sepsis [6,9,16]. Apart from lymphocytes, pro-adrenomedullin and the urokinase plasminogen activator receptor (uPAR) are the most promising prognostic markers to date. Furthermore, monocytic HLA-DR expression may also serve as a marker for sepsis-induced immunosuppression [6,9,16].

Neuropilin-1 (NRP-1), which has been extensively studied in neurons and axon guidance, is a transmembrane glycoprotein that acts as a coreceptor for several extracellular ligands, including Semaphorin family members (Class III and IV), certain isoforms of vascular epithelial growth factors (VEGF), and transforming growth factor beta (TGF- β) [17,18,19]. These ligands are involved in the regulation of cell apoptosis, cell migration, tumor suppression, angiogenesis, and immune regulation [17,20]. In humans, NRP-1 is expressed on dendritic cells, the arterial endothelium, and a subset of regulatory T cells in lymphoid tissue [18,21,22,23,24]. NRP-1 has been described as a mediator of contacts between dendritic cells and T lymphocytes via homotypic interactions. It plays a role in the initiation of the primary immune response, leading, e.g., to prolonged dendritic cell-to-T-cell interactions [18,20,23,25,26]. NRP-1 mediates those cell-to-cell contacts by acting as a coreceptor through coupling with, for example, the Semaphorin 3A receptor via the CUB domain. This binding locks Plexin A and Semaphorin 3A together, enhancing signal transduction and Semaphorin 3A activity and preventing NRP-1 localization, leading to the disruption of dendritic cell-to-T-cell contacts [17,27]. Proposed functions include the induction of T cell anergy by the aforementioned mechanism and the secretion of Interleukin-10 (IL-10) through the interaction with Semaphorin 3A. Additional functions include the suppression of tissue-specific immune responses through enhanced affinity for vascular endothelial growth factor 2 (VEGF2), the direct suppression of T effector cells, and the conversion of NRP-1-expressing CD4^+^ T cells into T regulatory cells by enhancing the binding of TGF-β [17]. Unlike in mice, NRP-1 blockade in humans has not been found to significantly affect immunosuppression [17]. There are several soluble isoforms of NRP-1 (sNRP-1) that lack the transmembrane or cytoplasmic domains, which enables them to bind NRP-1 ligands and act as their antagonists, inhibiting NRP-1 activity [19,28,29,30]. Measurement of the total soluble NRP-1 has become available using a novel sandwich ELISA [31]. However, the extent of sNRP-1 functions remains incompletely understood [17,32,33,34].

Recently, NRP-1 has been identified as a facilitator of cell entry and infectivity in SARS-CoV-2 infection and COVID-19. It has been proposed as a potential therapeutic target [35,36,37,38,39]. NRP-1 has been extensively researched as a prognostic biomarker and therapeutic target in cancer and leukemia [17,19,24,25,28,33,34]. Interestingly, NRP-1 has also been found to be expressed in liver cells and appears to be upregulated in various liver-related pathologies [39,40,41]. Other diseases that have been studied include kidney injury [42,43,44,45], autoimmune disease [46,47], and neurological disorders [48,49]. However, there is a lack of data on NRP-1 in human sepsis, despite efforts to understand its role in sepsis and its connection to kidney failure [50,51,52,53,54,55,56]. Given the involvement of NRP-1 in the immune system and regulation of vascular permeability [22,32,57,58], this study aims to investigate the role of its antagonist, sNRP-1, in critical illness and sepsis and assess its potential as a biomarker in this setting.

## 2. Results

### 2.1. Serum Levels of Soluble Neuropilin-1 Are Elevated in Critical Illness and Sepsis

One hundred and eighty patients were enrolled in the study between 2006 and 2011, of whom one hundred and twenty were diagnosed with sepsis. As a healthy control group, forty-eight patients were enrolled. The median age of the patient cohort was 65 years, with no difference between septic and nonseptic patients. Furthermore, there was no difference in the distribution of sex, comorbidities (measured by the Charlson Comorbidity Index, CCI), the need for mechanical ventilation, or short-term mortality (30 days) between the patient cohorts. However, patients with sepsis compared to patients without sepsis showed higher disease severity (APACHE II score, median of 19 vs. 14 points, *p* < 0.001), more severe organ failure (SOFA score, median of 9 vs. 7.5, *p* = 0.044), had a higher vasopressor demand (75.7% vs. 50.9%, *p* = 0.002), and thus stayed longer in the intensive care unit (ICU) (median of 9.5 vs. 6 days, *p* = 0.005). While ICU mortality was higher in septic patients compared to nonseptic patients (25 vs. 10%, *p* = 0.005), there was no difference in mortality at 30 days (29.8% vs. 20%, *p* = 0.243). However, long-term, one-year mortality was higher (67.9% vs. 37.5%, *p* = 0.003) (Table 1).

**Figure 1:**
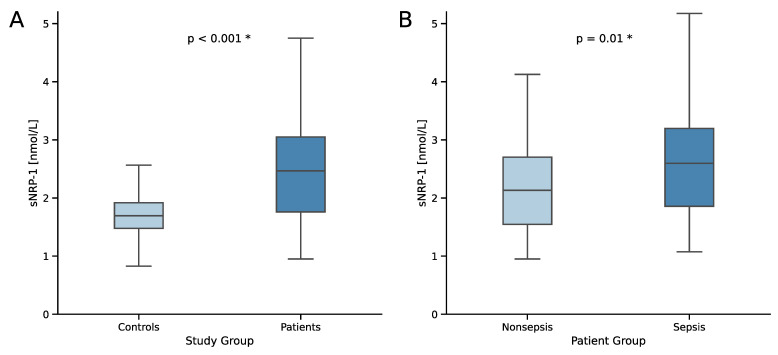
Serum sNRP-1 concentrations in (**A**) controls compared to critically ill patients and (**B**) comparison between critically ill patients with and without sepsis. Sample sizes: controls *n* = 48, patients *n* = 180, nonsepsis *n* = 60, sepsis *n* = 120. Significance between groups was assessed using the Mann–Whitney U test. *p*-values < 0.05 were considered statistically significant and were highlighted (*).

Median serum concentrations of soluble Neuropilin-1 (sNRP-1) were higher in the patient cohort compared to healthy controls (2.47 vs. 1.70 nmol/L, *p* < 0.001; Figure 1A). Importantly, we observed even higher concentrations of sNRP-1 in septic patients compared to those without (2.60 vs. 2.13 nmol/L, *p* = 0.01; Figure 1B). After adjusting for disease severity and organ failure (using SOFA and APACHE II scores) as potential confounders and employing propensity score matching, these differences remained significant (sNRP-1 median of 2.67 in septic vs. 2.13 nmol/L in nonseptic patients in matched cohort, *p* = 0.02).

### 2.2. Soluble Neuropilin-1 Levels Are Independent of Age and Disease Categories in Critical Illness, but Altered in Chronic Organ Failure

In order to examine other possible regulations and influencing factors of sNRP-1 levels in critical illnesses, we investigated whether chronic disease conditions have an influence on sNRP-1 serum concentrations. In a regression analysis, liver disease, chronic kidney failure, and the Charlson Comorbidity Index (CCI) showed statistically significant coefficients. However, in a multivariable model, only the significance of chronic kidney failure was retained (Table 2). In a subgroup analysis, we observed higher sNRP-1 levels in patients with known chronic kidney disease (median of 2.92 nmol/L, *p* < 0.001). Interestingly, patients with liver disease and chronic heart failure also showed a tendency toward higher concentrations of sNRP-1. On the other hand, patients with chronic obstructive lung disease had a tendency towards lower serum levels of sNRP-1, although these observations did not reach statistical significance (Table A1).

The most common cause of sepsis in the study cohort was a pulmonary site of infection (56.7%), followed by other infections such as bloodstream or skin infections (20.8%), and abdominal infections (15%). We did not observe a difference in sNRP-1 serum concentrations between the different causes of sepsis (*p* = 0.141). Patients with nonseptic critical illnesses were admitted due to cardiocirculatory disorders (23.3%), respiratory failure (20.0%), liver disease (16.7%), and various other diseases (40.0%). When comparing causes of nonseptic patients, we also could not find a difference in sNRP-1 levels (*p* = 0.122; Table 3).

Further examination of the influence of demographics on the serum levels of sNRP-1 revealed that male patients exhibited higher serum concentrations of sNRP-1 (median of 2.63 vs. 2.17 nmol/L, *p* = 0.034). However, age or body mass index (BMI) did not correlate with serum sNRP-1 in critically ill patients (Spearman’s r 0.085, *p* = 0.258 and Spearman’s r 0.089, *p* = 0.237 respectively; Table 4).

### 2.3. Serum Concentrations of Soluble Neuropilin-1 Correlate with Organ Failure in Critical Illness and Sepsis

Extensive correlation analyses were conducted to provide insight into the potential regulation of sNRP-1 in critically ill patients. With regard to peripheral blood counts and markers of inflammation, moderate to strong positive correlations were observed with C-reactive Protein (CRP) and procalcitonin, but not with Interleukin-6 or Interleukin-10. Most notably, multiple moderate to strong correlations were observed concerning laboratory kidney function, with positive correlations of sNRP-1 (potassium, urea, creatinine, estimated glomerular filtration rate, and cystatin C). Additionally, the days of renal replacement therapy in the ICU positively correlated with sNRP-1. After adjusting for chronic kidney disease using multiple linear regression, creatinine (Regression coefficient 0.07, CI 95% 0.01–0.14, *p* = 0.022), and cystatin C (Regression coefficient 0.19, CI 95% 0.06–0.32, *p* = 0.004) remained independently associated with higher sNRP-1 serum concentrations in critical illness. Furthermore, several noteworthy correlations were identified with impaired liver and coagulator function. In this instance, the international normalized ratio (INR) and activated partial thromboplastin time (aPTT) showed a medium-strength correlation with sNRP-1. Moreover, total bilirubin and gamma-glutamyl transpeptidase, but not alanine or aspartate aminotransferases (ALT, AST), showed positive correlations with serum sNRP-1 concentrations. Of interest, a positive correlation was also observed between N-terminal pro-B-type natriuretic peptide and sNRP-1 (NTproBNP), but not with vasopressor demand. Moreover, patients with lower PaO_2_/FiO_2_ ratios showed higher levels of sNRP-1. However, the number of days on mechanical ventilation showed no correlation with sNRP-1. In line with this, we observed higher sNRP-1 serum levels in patients with higher disease severity (APACHE II score) and more organ failure (SOFA score) (Table 4).

### 2.4. Elevated Soluble Neuropilin-1 Serum Levels Are Associated with Long-Term Mortality in Critical Illness and Sepsis, Dependent on Acute and Chronic Organ Dysfunction

**Figure 2:**
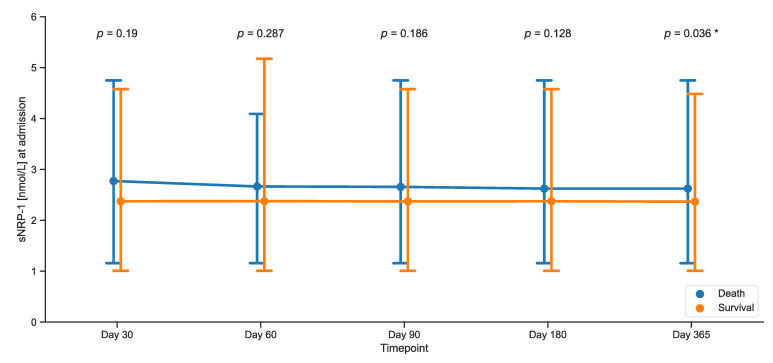
Serum sNRP-1 levels at ICU admission and survival status at consecutive timepoints. The sample size for this study consisted of 180 patients. Vertical bars represent the lower and upper interquartile range multiplied with 1.5. Significance between groups was assessed using the Mann–Whitney U test. *p*-values < 0.05 were considered statistically significant and were highlighted with an asterisk (*).

**Figure 3:**
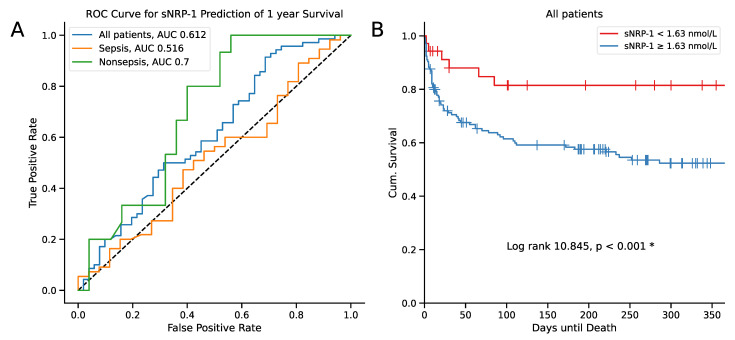
(**A**) Receiver Operating Characteristic (ROC) curves for predicting one-year survival based on serum sNRP-1 levels in all patients, as well as in sepsis and nonsepsis patients. The Kaplan–Meier curves in (**B**) represent serum sNRP-1 levels below 1.63 nmol/L (red) and equal to or above 1.63 nmol/L (blue) in all patients. Censored events are marked by a vertical line. The cutoff value for the Kaplan–Meier curve was determined using the Youden index. The sample sizes were as follows: total patients *n* = 180, nonsepsis *n* = 60, sepsis *n* = 120. Significance between groups was evaluated using the log rank test. *p*-values < 0.05 were considered statistically significant and were highlighted (*). Abbreviation: AUC: Area Under Curve.

Next, we investigated the impact of serum sNRP-1 on survival. Initially, the serum concentrations of sNRP-1 at admission to the ICU were compared between surviving and deceased patients at different time points (i.e., 30, 60, 90, 180, and 365 days after ICU admission). We found no difference in sNRP-1 concentrations at admission when comparing the early timepoints (i.e., 30, 60, 90, and 180 days after ICU admission; Figure 2). However, lower sNRP-1 serum concentrations at ICU admission were observed in surviving patients at one year (*p* = 0.036, Figure 2). When comparing survivors to non-survivors 1 year after ICU admission, non-survivors were of an older age, showed a higher extent of acute organ failure, and had more frequent chronic disease (Table 5). However, after adjustment for covariates as predictors of one-year survival (age, APACHE II score, SOFA score, and CCI), sNRP-1 was no longer an independent predictor of survival (Table A2). To determine the optimal cutoff of sNRP-1 serum concentration for predicting one-year survival, we used the Youden index and found it to be at 1.63 nmol/L. We then calculated receiver operating characteristic curves (ROC) and their corresponding area under the curve (AUC) to evaluate the prognostic capabilities of sNRP-1 (Figure 3A). The AUC for predicting one-year survival in all patients was 0.612 (95% CI 0.509–0.716), while it was 0.516 in septic patients and 0.7 in nonseptic critically ill patients (Figure 3A). In a subsequent Kaplan–Meier curve analysis, we observed the greatest separation between curves at later timepoints (Log-rank 10.845, *p* < 0.001; Figure 3B). Using the described cutoff of 1.63 nmol/L for prediction of survival at one year after ICU admission yields a sensitivity of 91.4% and a specificity of 30.7%, resulting in a positive predictive value of 64.6% and a negative predictive value of 72.7% (Table 6). To facilitate comparison to known predictors of survival in the critical care setting, we also conducted ROC analyses for the SOFA and APACHE II scores. Here, the SOFA score showed an AUC of 0.689 (95% CI 0.592–0.786) and the APACHE II score an AUC of 0.725 (95% CI 0.630–0.820) with all confidence intervals including sNRP-1 overlapping (Table 7).

## 3. Discussion

Given its involvement in the immune system, regulation of vascular permeability, tumor promotion and progression, the clinical impact of NRP-1 has been extensively studied in cancer, leukemia, autoimmune disease, liver pathologies, kidney injury, and COVID-19. These studies have explored its clinical impact in terms of pathophysiology, biomarker potential in disease, and as a potential target for cancer therapy [20,35,36,37,38,39,40,41,42,43,44,45,46,47,48,49,50,51,52,53,54,55,56,58]. To our knowledge, this is the first study investigating the serum levels of its soluble form (sNRP-1), which acts as an antagonist to NRP-1, in critical illness and sepsis. We not only found elevated levels of sNRP-1 in critical illness, but even an upregulation of those levels in patients with sepsis, independent from disease severity and organ failure. Moreover, we found increased serum levels of sNRP-1 in organ failure, most pronounced in kidney and hepatic failure. Of note, the disease category among septic and nonseptic patients did not show an impact on sNRP-1 serum concentrations in our cohort. Finally, we found that serum sNRP-1 levels were lower upon admission in patients who ultimately survived in the long term. These findings suggest that sNRP-1 may play a role in the pathophysiology of critical illness and sepsis, as well as in the mechanisms of organ failure, potentially acting as a driver of mortality.

While NRP-1 has been extensively studied in mice and to a lesser extent in humans, particularly in the context of neuronal guidance, cancer, and other diseases, the role of its soluble form (sNRP-1) as an antagonist of NRP-1 remains unclear. Previous research has provided some data on the role of sNRP-1 in breast and ovarian cancer, as well as in preeclampsia [19,32,33,34]. Encouraging its use as a biomarker, we did not observe a correlation between sNRP-1 levels and age or body mass index (BMI; Table 4), but we did find higher levels of sNRP-1 in male patients, suggesting the possible need for sex-specific cutoffs. However, despite demonstrating high sensitivity, the specificity is rather low, resulting in mediocre negative and positive predictive values (Table 6). Additionally, we were unable to prove the superiority of sNRP-1 as a predictor of survival compared to other established predictors like the SOFA or APACHE II scores (Table 6, Table 7 and Table A2). Consequently, the role of sNRP-1 as a predictive biomarker in critical illness requires further evaluation and cannot be recommended at this time.

Current research suggests a role for sNRP-1 in regulating the primary immune response, vascular permeability, and angiogenesis [29,57]. NRP-1 facilitates the regulation and suppression of T cells by inducing T cell anergy, suppressing T effector cells, and converting them into T regulatory cells [18,23,24]. As an antagonist of NRP-1, sNRP-1 may inhibit the suppression of the primary immune response and thus stimulate it. Supporting this theory, we observed higher serum levels of sNRP-1 in critical illness and sepsis (Figure 1), which are disease states that generally activate the immune system. Of note, this was independent of organ failure and disease severity in a matched cohort, considering that sNRP-1 positively correlates with SOFA and APACHE II scores in the study cohort (Table 4). Moreover, we did not find a difference in sNRP-1 levels between the disease categories of (non)septic disease (Table 3).

Previously, NRP-1 has been associated with liver pathologies [39,41] and kidney disease [42,43,44,45,50,52,53]. Urinary NRP-1 has been proposed as a biomarker for lupus nephritis [42] due to its high expression in mesangial cells. Furthermore, NRP-1 has been found to play a role in the inflammatory response in acute kidney injury in a mouse model [52]. In addition, the Semaphorin 4A-Neuropilin-1 axis was found to alleviate the development of ischemia–reperfusion injury by facilitating the stability and function of T regulatory cells in mice [43]. Concerning the liver, inhibition of NRP-1 has been shown to improve steatotic liver disease in obese mice [40]. In our study, we also found higher levels of sNRP-1 in patients with chronic kidney disease (Table 2), in patients with elevated laboratory markers of kidney injury and impaired kidney function (Table 4), as well as in patients with impaired liver function (Table 4) in the context of critical illness. This suggests a possible involvement of sNRP-1 in the pathophysiology of these disorders. Ultimately, this also leads to a correlation between sNRP-1 and overall organ failure in critical illness, as measured by the SOFA score (Table 4).

To date, elevated levels of sNRP-1 have been linked to more advanced ovarian cancer [33] and have been identified as an independent marker of poor prognosis in early breast cancer [34]. In this study, we observed higher levels of sNRP-1 upon admission to the ICU in patients who did not survive one year after ICU admission (Figure 2). However, this was more pronounced in critically ill patients without sepsis (Figure 3A). As critically ill patients with sepsis already exhibited elevated sNRP-1 levels independent of organ failure, this effect appears to prevent a distinction between death and survival in this group. In nonseptic critically ill patients, however, organ failure may determine sNRP-1 levels, allowing for discrimination between death and survival dependent on organ failure in that patient group. This aligns with the broader understanding of disease progression and higher mortality in patients with elevated sNRP-1.

Some limitations should be openly discussed to facilitate the interpretation of the study. We conducted a single-center study that recruited patients from a single medical ICU, which limits the generalizability of the results. Additionally, since the study period predates the implementation of the Sepsis-3 criteria, we had to retrospectively apply those criteria to fit the current definition of sepsis. Conducting further multi-center prospective studies would likely enhance the generalizability and understanding of the value of sNRP-1 in critical illness. Due to the extensive statistical approach, especially in our correlation analyses, false-positive results are to be expected and should be carefully interpreted within the clinical context. Furthermore, the septic patients in our study cohort exhibited more frequent and severe organ failure, as measured by the SOFA score, which could impact sNRP-1 levels. Thankfully, we were able to account for these covariates by using a matched cohort (correcting for SOFA and APACHE II scores) and applying multivariate regression models. However, due to the design of our study, we cannot make deductions about the source or the release mechanisms and pathophysiology of sNRP-1 in critical illness and sepsis. The study, which was the first of its kind to investigate the value of sNRP-1 in critical illness and sepsis, yielded several interesting results pointing towards higher sNRP-1 serum levels in sepsis and organ failure.

## 4. Materials and Methods

### 4.1. Study Design

**Figure 4:**
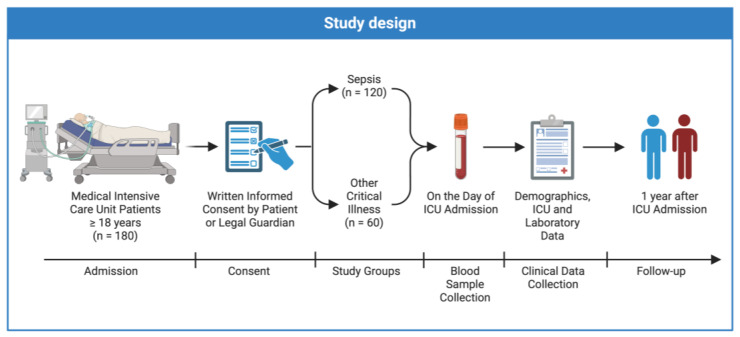
Graphical representation of the study design.

This study was conducted as an observational cohort study to investigate the role of soluble Neuropilin-1 (sNRP-1) in critically ill and septic patients. We recruited 180 patients prospectively from the medical intensive care unit (ICU) of the Department of Gastroenterology, Digestive Disease, and Intensive Care Medicine at the University Hospital RWTH Aachen. Written informed consent was obtained from the patient, their spouse, or an appointed legal guardian. Patients above the age of 18 with available blood samples on the day of ICU admission were included, as previously described [59,60,61,62]. Exclusion criteria included expected short-term ICU treatment (<48 h), admission from another ICU, acute poisoning, and pregnancy. For discrimination between septic and non-septic patients upon ICU admission, the Third International Consensus Criteria Definition for Sepsis was applied [2]. The comorbidities of patients were assessed using the Charlson Comorbidity Index [63]. Upon consent, we contacted the patient, their relatives, or their primary care physician to collect follow-up data. We collected blood samples from 48 healthy volunteers from our local blood bank with normal values for blood counts to serve as a control group. Prior to inclusion, healthy volunteers were subjected to a clinical examination to exclude acute infections or chronic diseases. This study was conducted in accordance with the 1964 Declaration of Helsinki and was approved by our local ethics committee (EK150/06) of the University Hospital RWTH Aachen (Figure 4).

### 4.2. Soluble Neuropilin-1 Measurements

Blood samples were collected on the day of admission to the ICU and centrifuged at 4 °C for 10 min. Serum aliquots were kept frozen at −80 °C until further investigation. Concentrations of soluble Neuropilin-1 (sNRP-1) were analyzed in March 2022 using a commercially available enzyme-linked immunosorbent assay (ELISA) kit, following the manufacturer’s instructions (cat. no. BI-20409, Biomedica Medizinprodukte GmbH, Divischgasse 4, Vienna, Austria). The measurements were conducted without knowledge of the clinical or other laboratory data of the study subjects.

### 4.3. Statistical Analysis

Analysis and visualization of data were performed using SPSS Version 29 (SPSS, Chicago, IL, USA) and the following packages: NumPy version 1.21.5 [64], Pandas version 1.4.4 [65], Matplotlib version 3.5.2 [66], Seaborn version 0.11.2 [67], Pingouin version 0.5.3 [68], Scikit-learn version 1.0.2 [69], and Lifelines version 0.27.7 [70] in Jupyter Notebooks version 6.5.4 [71] with Python version 3.11 [72]. Data were presented as median and range due to the skewed distribution of most parameters. A significance level of *p* = 0.05 was used for all statistical calculations. The two-tailed Mann–Whitney U test or chi-squared test was used to compare two ungrouped samples when a normal distribution could not be assumed. The Kruskal–Wallis test was applied for comparisons involving more than two groups. Propensity scores were used to match cases for the purpose of adjustment for covariates. Correlations between parameters were assessed by Spearman’s rank correlation test. Single and multiple linear and logistic regression models were used to detect and adjust for covariates after correlation analysis. Patient survival was graphed using Kaplan–Meier curves, and significance was determined using the log rank test. Optimal cut-off values for parameters were calculated using the Youden index (sum of sensitivity and specificity minus one). The predictiveness of markers was evaluated by graphing receiver operating characteristic (ROC) curves and calculating the corresponding area under the curve (AUC). A comparison between the AUC of ROC was performed using DeLong’s test.

## 5. Conclusions

Critically ill and septic patients exhibit higher levels of circulating sNRP-1, which also correlates with organ failure, particularly hepatic and kidney function impairment. Interestingly, long-term survivors have lower levels of sNRP-1 upon admission to the ICU. Future research should focus on validating these findings in larger and more diverse groups, as well as understanding the pathophysiology, source, and mechanisms underlying the release of sNRP-1.

## Figures and Tables

**Table 1:** Baseline patient characteristics.

Parameter	All Patients	Nonsepsis	Sepsis	*p*-Value
Number n	180	60	120	
Sex (female/male) n	73/107	21/39	52/68	0.362
Age (years)	65 (18–90)	63.5 (18–85)	65.5 (20–90)	0.911
APACHE II score	17.5 (2–43)	14 (2–33)	19 (4–43)	<0.001 *
SOFA score	9 (0–20)	7.5 (0–17)	9 (2–20)	0.044 *
Charlson Comorbidity Index	5 (0–15)	5 (0–13)	5 (0–15)	0.397
Liver disease n (%)	11 (6.1)	7 (11.7)	4 (3.3)	0.061
Chronic kidney failure n (%)	45 (25.0)	12 (20.0)	33 (27.5)	0.361
Mechanical ventilation n (%)	124 (69.3)	40 (66.7)	84 (70.6)	0.715
Vasopressor demand n (%)	113 (67.3)	29 (50.9)	84 (75.7)	0.002 *
ICU days n	7 (1–83)	6 (1–71)	9.5 (1–83)	0.005 *
Death in ICU n (%)	36 (20.0)	6 (10.0)	30 (25.0)	0.030 *
30-day mortality n (%) ^#^	45 (26.6) ^#^	11 (20.0) ^#^	34 (29.8) ^#^	0.243
sNRP-1 (nmol/L)	2.47 (0.95–5.67)	2.13 (0.95–5.67)	2.60 (1.07–5.17)	0.010 *

The median and range (in parentheses) are given, unless indicated otherwise. Abbreviations: APACHE: acute physiology and chronic health evaluation; SOFA: sequential organ failure assessment; ICU: intensive care unit; sNRP-1: soluble Neuropilin-1. Significance between septic and nonseptic patients was assessed using the Mann–Whitney U test or chi-squared test, respectively. *p*-values < 0.05 were considered statistically significant and were highlighted (*). ^#^ Including cases of death in ICU within the first 30 days. Lost to follow-up within the first 30 days: all patients: *n* = 11, nonsepsis: *n* = 5; sepsis: *n* = 6.

**Table 2:** Uni- and multivariable regression analysis for covariates of sNRP-1.

	Univariable Regression			Multivariable Regression		
Covariate	Coefficient	95% CI	*p*	Coefficient	95% CI	*p*
Age	0.006	−0.002–0.015	0.150			
BMI	0.001	−0.013–0.015	0.015			
Sex	0.235	−0.040–0.510	0.093			
Diabetes	0.144	−0.151–0.438	0.337			
Liver Disease	0.565	0.004–1.126	0.049 *	0.526	−0.016–1.069	0.057
Coronary Artery Disease	0.146	−0.146–0.437	0.325			
Hypertension	0.094	−0.182–0.370	0.503			
Chronic Alcohol Abuse	0.206	−0.208–0.620	0.328			
Chronic Obstructive Lung Disease	−0.321	−0.641–0.000	0.050			
Active Malignancy	0.144	−0.263–0.550	0.486			
Chronic Heart Failure	0.227	−0.071–0.525	0.135			
Chronic Kidney Failure	0.647	−0.348–0.946	<0.001 *	0.616	0.317–0.915	<0.001 *
Charlson Comorbidity Index	0.053	0.013–0.093	0.010 *	0.033	−0.007–0.072	0.105

* Significance was assessed using a linear regression model.

**Table 3:** Disease etiology of the study population and subgroup sNRP-1 concentrations.

Etiology of (Non-)Septic Critical Illness	Sepsis *n* = 120, *n* (%)	Nonsepsis *n* = 60, *n* (%)	sNRP-1 (nmol/L)	*p*-Value
Pulmonary	68 (56.7)		2.54 (1.07–4.75)	0.141
Abdominal	18 (15.0)		2.67 (1.15–5.17)	
Urogenital	9 (7.5)		2.60 (1.47–4.58)	
Other	25 (20.8)		2.91 (1.64–4.48)	
Cardiocirculatory disorder		14 (23.3)	2.28 (1.02–5.67)	0.122
Respiratory failure		12 (20.0)	1.95 (1.05–3.03)	
Advanced liver disease		10 (16.7)	2.59 (2.00–5.11)	
Other		24 (40.0)	1.76 (0.95–4.12)	

The absolute numbers and percentage of the respective subgroup (in parentheses) or median and range (in parentheses) are given. Significance between more than two groups was assessed using the Kruskal–Wallis test.

**Table 4:** Correlations of clinical and laboratory parameters with sNRP-1 serum concentrations at ICU admission.

Parameter	Spearman’s r	*p*-Value
Demographics		
Age	0.085	0.258
Body mass index	0.089	0.237
Blood count and markers of inflammation		
Leukocytes	0.129	0.084
Hemoglobin	−0.202	0.007 *
Platelets	−0.127	0.089
C-reactive protein	0.384	<0.001 *
Procalcitonin	0.338	<0.001 *
Interleukin 6	0.118	0.172
Interleukin 10	0.088	0.434
Electrolytes and renal system		
Sodium	−0.042	0.576
Potassium	0.192	0.01 *
Uric acid	0.076	0.369
Urea	0.391	<0.001 *
Creatinine	0.356	<0.001 *
eGFR	−0.339	<0.001 *
Cystatin C	0.436	<0.001 *
Renal replacement therapy days	0.306	<0.001 *
Hepato-pancreatic-biliary system and coagulation		
Protein, total	−0.171	0.037 *
Albumin	−0.15	0.154
INR	0.229	0.002 *
aPTT	0.246	0.001 *
Bilirubin, total	0.257	0.001 *
γGT	0.295	<0.001 *
AST	0.025	0.75
ALT	0.045	0.552
Lipase	−0.182	0.031 *
Cardiopulmonary system		
NTproBNP	0.362	<0.001 *
Norepinephrine demand	0.078	0.314
Horovitz quotient (PaO_2_/FiO_2_)	−0.236	0.03 *
FiO_2_	0.173	0.111
Days of mechanical ventilation	0.087	0.246
Metabolism		
Glucose	−0.149	0.047 *
HbA1c	0.042	0.76
Insulin	−0.277	0.049 *
C-Peptide	−0.08	0.579
Cholesterol, total	−0.128	0.132
HDL-cholesterol	−0.24	0.087
LDL-cholesterol	−0.218	0.124
Triglycerides	0.085	0.317
Disease severity		
Length of stay in hospital	0.21	0.005 *
Length of stay on ICU	0.127	0.090
SOFA score on admission	0.193	0.01 *
SOFA score after 48 h	0.174	0.105
APACHE II score	0.201	0.009 *

Abbreviations: eGFR: estimated Glomerular filtration rate; INR: International normalized ratio; aPTT: activated partial thromboplastin time; γGT: Gamma-glutamyl transpeptidase; AST: Aspartate aminotransferase; ALT: Alanine aminotransferase; NTproBNP: N-terminal pro B-type natriuretic peptide; FiO_2_: Fraction of inspired oxygen; PaO_2_: arterial oxygen partial pressure; HbA1c: Glycosylated hemoglobin A1; HDL: high-density lipoprotein; LDL: low-density lipoprotein; ICU: intensive care unit; SOFA: Sequential organ failure assessment; APACHE II: acute physiology and chronic health evaluation II. Spearman rank correlation test was used to calculate significant correlations of positive and negative nature. *p*-values < 0.05 were considered statistically significant and were highlighted (*).

**Table 5:** Comparison between long-term survivors and non-survivors 1 year after ICU admission.

Parameter	All Patients	Survivors	Non-Survivors	*p*-Value
Number n	121	51	70	
Sepsis n (%)	81 (66.9)	26 (51.0)	55 (78.6)	0.002 *
Sex (female/male) n	44/77	20/31	24/46	0.715
Age (years)	66 (12–90)	60 (18–81)	69 (22–90)	<0.001 *
BMI (kg/m^2^)	25.8 (15.3–62.3)	26.2 (15.3–46.3)	25.4 (16.4–43.0)	0.430
APACHE II score	18 (2–43)	12 (2–28)	20 (5–43)	<0.001 *
SOFA score	9 (0–20)	6 (0–17)	10 (2–20)	<0.001 *
Charlson Comorbidity Index	5 (0–15)	3 (0–10)	6 (0–15)	<0.001 *
Liver disease n (%)	8 (6.6)	5 (9.8)	3 (4.3)	0.403
Chronic kidney failure n (%)	30 (24.8)	13 (25.5)	17 (24.3)	0.951
Mechanical ventilation n (%)	83 (68.6)	29 (56.9)	54 (77.1)	0.02 *
Vasopressor demand n (%)	73 (60.3)	26 (51.0)	50 (71.4)	<0.001 *
ICU days n	7 (1–71)	7 (1–56)	7 (1–71)	0.996
Death in ICU n (%)	36 (29.8)	0 (0)	36 (51.4)	<0.001 *
1-year mortality n (%)	70 (57.9)	0 (0)	70 (100)	<0.001 *
sNRP-1 (nmol/L)	2.55 (1.01–5.67)	2.37 (1.01–5.67)	2.62 (1.16–5.17)	0.036 *

The median and range (in parentheses) are given, unless indicated otherwise. Abbreviations: APACHE: acute physiology and chronic health evaluation; SOFA: sequential organ failure assessment; ICU: intensive care unit; sNRP-1: soluble Neuropilin-1. Significance between septic and nonseptic patients was assessed using the Mann–Whitney U test or chi-squared test, respectively. *p*-values < 0.05 were considered statistically significant and were highlighted (*).

**Table 6:** Test statistics for sNRP-1 prediction of survival at 1 year after ICU admission.

Statistic	Value (%)
Sensitivity	91.4
Specificity	30.7
PPV	64.6
NPV	72.7

Abbreviations: PPV: positive predictive value, NPV: negative predictive value.

**Table 7:** Comparison of predictors of survival to sNRP-1.

Predictor of Survival at 1 Year	AUC	95% CI	*p*-Value
sNRP-1	0.612	0.509–0.716	
SOFA score	0.689	0.592–0.786	0.210
APACHE II score	0.725	0.630–0.820	0.103

Abbreviations: AUC: area under the curve in a receiver operating characteristics curve; CI: confidence interval. Significance between the AUCs was assessed using the DeLong’s test. *p*-values denote the comparison to the AUC of sNRP-1. *p*-values < 0.05 were considered statistically significant.

## Data Availability

The original data sets presented in this study are available on request from the corresponding author.

## Appendix A

**Table A1:** Comorbidities and their influence on sNRP-1 serum levels.

Comorbidity, *n*	sNRP-1 (nmol/L), Median (Range)	*p*-Value
Diabetes (*n* = 55)	2.63 (1.05–4.75)	0.169
Liver disease (*n* = 11)	2.87 (2.00–4.48)	0.051
Coronary artery disease (*n* = 57)	2.63 (1.02–4.48)	0.145
Hypertension (*n* = 74)	2.61 (0.95–5.17)	0.340
Chronic alcohol abuse (*n* = 22)	2.62 (1.20–4.36)	0.340
Chronic obstructive lung disease (*n* = 41)	2.10 (1.07–3.76)	0.078
Active malignancy (*n* = 23)	2.37 (1.67–4.75)	0.399
Chronic heart failure (*n* = 52)	2.65 (1.16–5.67)	0.081
Chronic kidney failure (*n* = 45)	2.92 (1.51–5.17)	<0.001 *

The median and range (in parentheses) are given, unless indicated otherwise. Significance between groups was assessed using the Mann–Whitney U test. *p*-values < 0.05 were considered statistically significant and were highlighted (*).

**Table A2:** Multivariable logistic regression analysis for the prediction of 1-year survival by sNRP-1 serum levels and covariates.

Parameter	OR (95% CI)	*p*-Value
Age	1.02 (0.98–1.05)	0.296
APACHE II score	1.06 (0.99–1.13)	0.063
SOFA score	1.13 (1.01–1.26)	0.038 *
Charlson Comorbidity Index	1.16 (0.97–1.39)	0.100
sNRP-1	1.13 (0.65–1.95)	0.674

Abbreviations: OR: odds ratio; CI: confidence interval. Significance was assessed using a multivariable logistic regression model. *p*-values < 0.05 were considered statistically significant and were highlighted (*).
